# The intriguing Cyclophilin A-HIV-1 Vpr interaction: prolyl *cis*/*trans *isomerisation catalysis and specific binding

**DOI:** 10.1186/1472-6807-10-31

**Published:** 2010-10-04

**Authors:** Sara M Solbak, Tove R Reksten, Victor Wray, Karsten Bruns, Ole Horvli, Arnt J Raae, Petra Henklein, Peter Henklein, Rene Röder, David Mitzner, Ulrich Schubert, Torgils Fossen

**Affiliations:** 1 From the Department of Chemistry, University of Bergen, N-5007 Bergen, Norway; 2 Centre of Pharmacy, University of Bergen, N-5007 Bergen, Norway; 3 Department of Structural Biology, Helmholtz Centre for Infection Research, D-38124 Braunschweig, Germany; 4 Department of Molecular Biology, University of Bergen, N-5020 Bergen, Norway; 5 Institute of Biochemistry, Charité Universitätsmedizin-Berlin, D-10117 Berlin, Germany; 6 Institute of Virology, University of Erlangen-Nürnberg, D-91054 Erlangen, Germany

## Abstract

**Background:**

Cyclophilin A (CypA) represents a potential target for antiretroviral therapy since inhibition of CypA suppresses human immunodeficiency virus type 1 (HIV-1) replication, although the mechanism through which CypA modulates HIV-1 infectivity still remains unclear. The interaction of HIV-1 viral protein R (Vpr) with the human peptidyl prolyl isomerase CypA is known to occur *in vitro *and *in vivo*. However, the nature of the interaction of CypA with Pro-35 of N-terminal Vpr has remained undefined.

**Results:**

Characterization of the interactions of human CypA with N-terminal peptides of HIV-1 Vpr has been achieved using a combination of nuclear magnetic resonace (NMR) exchange spectroscopy and surface plasmon resonance spectroscopy (SPR). NMR data at atomic resolution indicate prolyl *cis*/*trans *isomerisation of the highly conserved proline residues Pro-5, -10, -14 and -35 of Vpr are catalyzed by human CypA and require only very low concentrations of the isomerase relative to that of the peptide substrates. Of the N-terminal peptides of Vpr only those containing Pro-35 bind to CypA in a biosensor assay. SPR studies of specific N-terminal peptides with decreasing numbers of residues revealed that a seven-residue motif centred at Pro-35 consisting of RHFPRIW, which under membrane-like solution conditions comprises the loop region connecting helix 1 and 2 of Vpr and the two terminal residues of helix 1, is sufficient to maintain strong specific binding.

**Conclusions:**

Only N-terminal peptides of Vpr containing Pro-35, which appears to be vital for manifold functions of Vpr, bind to CypA in a biosensor assay. This indicates that Pro-35 is essential for a specific CypA-Vpr binding interaction, in contrast to the general prolyl *cis*/*trans *isomerisation observed for all proline residues of Vpr, which only involve transient enzyme-substrate interactions. Previously suggested models depicting CypA as a chaperone that plays a role in HIV-1 virulence are now supported by our data. In detail the SPR data of this interaction were compatible with a two-state binding interaction model that involves a conformational change during binding. This is in accord with the structural changes observed by NMR suggesting CypA catalyzes the prolyl *cis/trans *interconversion during binding to the RHFP^35^RIW motif of N-terminal Vpr.

## Background

The 96 amino acid virion-associated multifunctional viral protein R (Vpr) [1,2] is encoded by primate lentiviruses, the human immunodeficiency viruses, types 1 and 2 (HIV-1/HIV-2), and simian immunodeficiency viruses (SIV). This accessory protein fulfils multiple functions in the viral life cycle including increase of viral replication in non-dividing host cells, induction of G2 cell-cycle arrest [3,4], apoptosis [5,6], and transduction through cell membranes [7]. Vpr facilitates transport of the pre-integration complex into the nucleus of non-dividing cells [8] and interacts with several cellular factors, including the human peptidyl prolyl isomerase CypA [9].

The interaction of HIV-1 Vpr with CypA is known to occur *in vitro *and *in vivo *[9-11]. In addition to the extensively studied interaction between CypA and HIV-1 capsid, that is crucial for viral replication [12,13], CypA was also reported to be significant for the *de novo *synthesis of Vpr, as the Vpr-mediated cell cycle arrest in HIV-1 infected T cells appeared to be eliminated in the absence of CypA activity [9]. However, more recently Ardon et al. [10] concluded that the interaction of Vpr with CypA is independent of the ability of Vpr to induce cell cycle arrest. Nonetheless, specific inhibitors of the prolyl *cis/trans *isomerase activity of CypA, such as cyclosporine A and SDZ-NIM811 inhibit HIV replication [11,14-18].

Previous structural studies of Vpr by 2D NMR spectroscopy in aqueous organic solvents provided evidence of proline *cis/trans *isomerism for the highly conserved N-terminal Pro-5, -10, -14 and -35 of Vpr [19]. In particular Pro-35 exhibited a relatively high proportion of the *cis *isomer under these solvent conditions (15% *cis *isomer content). This suggested prolyl *cis/trans *isomerisation may be important for the folding of the molecule. At pH 7 Vpr has a relatively random structure in aqueous solution but assumes a folded structure in a hydrophobic membranous environment [7,19]. This fact together with observation of considerable amounts of CypA in virions [20] prompted a study, using surface plasmon resonance (SPR) spectroscopy, of the interaction of Vpr with the prolyl *cis/trans *isomerase CypA [9]. A qualitative interaction was detected for N-terminal peptides containing Pro-35, indicating an essential role for this Pro residue, although this could not be quantified. Indeed the interaction of Vpr with CypA could not be confirmed under the solution conditions used. Hence, the nature of the interaction of CypA with Pro-35 of N-terminal Vpr has remained undefined.

The catalytic activity of CypA, as a peptidyl-prolyl *cis/trans *isomerase (PPiase), has previously been studied on short model peptides containing one Pro residue. Thus, based on interaction studies of eight short model peptides, each comprising four residues containing only one Pro residue preceded by a variety of different amino acids, Harrison and Stein [21] concluded that CypA exhibited a broad tolerance as a prolyl *cis/trans *isomerase of several substrates. Endrich et al. [22] reported that CypA interacts with three 14-37 residues HIV-1 Capsid-derived peptides, each containing 1-4 Pro residues and at least one Gly-Pro motif. However, exact information regarding the interaction of individual Pro residues within peptides containing more than one Pro unit was not accessible with the applied methodology.

The suitability of NMR spectroscopy for studying the catalytic activity of CypA has been demonstrated in a more limited number of reports. NMR spectroscopy is the only method wherefrom information about prolyl *cis/trans *isomerase interaction of CypA with individual Pro residues of a peptide, containing several Pro residues, is accessible at atomic resolution. However when determined, only selective interactions involving one of the Pro residues in the peptides [12,23] or the full length protein [13] containing more than one Pro residue have been reported.

In this paper the interactions of CypA with the N-terminal synthetic Vpr (*s*Vpr) peptides *s*Vpr^1-20^, *s*Vpr^21-40 ^and *s*Vpr^25-40^, containing all the highly conserved Pro residues of Vpr, have been studied by NMR spectroscopy at atomic resolution. The NMR experiments were performed in aqueous phosphate buffer at physiological pH 7, where CypA retains its enzymatic activity and Vpr exists in an unstructured state [7,19]. Complementary experiments, using SPR spectroscopy and NMR spectroscopy, have provided an unambiguous distinction between the substrate requirements for the catalytic activity of CypA and those for a strong specific binding to N-terminal Vpr. For the first time this has also allowed quantification of this interaction.

## Results

The combination and access to both highly pure recombinant CypA and synthetic N-terminal Vpr peptides, as well as sensitive NMR and SPR facilities, has allowed us to probe the interactions of Vpr and CypA in more detail than previously.

### Characterization of sVpr^1-20^, sVpr^25-40 ^and sVpr^21-40 ^by NMR spectroscopy

Previous studies [7,19,24,25] have shown that Vpr has a relatively random structure in aqueous solution at pH 7, but has a propensity for three well-defined α-helical structural domains in aqueous solution at lower pH or in the presence of organic co-solvent (TFE-*d2 *or CD_3_CN), where the extent of secondary structure is dependent on the hydrophobicity of the solvent.

**Figure 1:**
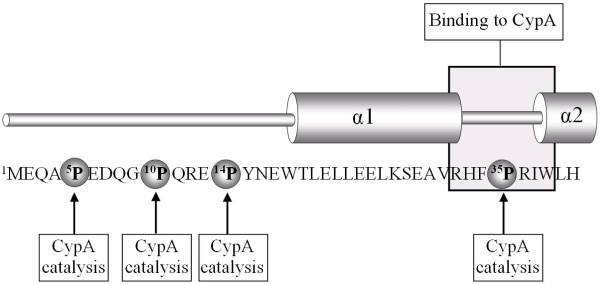
**Interactions of N-terminal Vpr with CypA**. Schematic overview of the N-terminal sequence of HIV-1_NL4-3 _Vpr showing its interactions with CypA that include regions undergoing transient enzymatic prolyl *cis/trans *catalysis and the region of strong selective binding. In aqueous phosphate buffer at pH 7 N-terminal Vpr peptides, as well as full-length Vpr, are essentially unstructured but assume α-helical structure at lower pH and under hydrophobic conditions [7,19]. Notice that the region of selective strong binding of Vpr with CypA includes the entire loop region connecting regions that form α-helices 1 and 2.

The N-terminal region of Vpr includes four highly conserved Pro residues at positions 5, 10, 14 and 35, respectively (Fig. 1) (more than 98% conserved according to references [19] and [26]). Among these, the presence of Pro-35 is vital for several functional interactions of Vpr. The tertiary structure of Vpr observed in aqueous and 30% aqueous acetonitrile solution at low pH is characterized by a hydrophobic core formed by the three α-helices [25] and partly reliant on the presence of the Pro-35 residue that acts as a helix breaker between helix 1 comprising residues 17-33, in which the p6 binding domain of Vpr is located [27], and helix 2 (comprising residues 38-50) of Vpr [28]. At pH 7 Vpr is essentially unstructured in aqueous solution and requires under these conditions a hydrophobic environment to achieve α-helical structure [7,19] (Additional file 1, Fig. S1).

**Figure 2:**
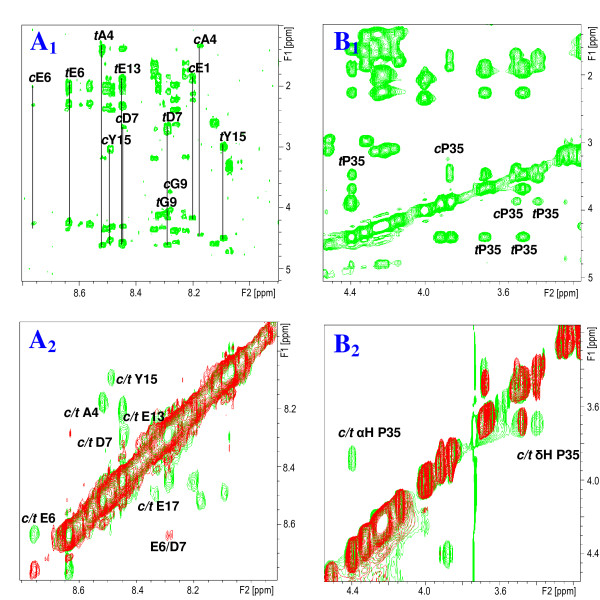
**Presence of *cis *and *trans *proline isomers in N-terminal *s*Vpr**. **A_1_**: Fingerprint region of the 2D ^1^H-^1^H TOCSY spectrum showing the assigned spin systems of residues close to proline residues in *s*Vpr(1-20) and **A_2_**: Superimposed expanded HN-HN regions of the 2D ^1^H-^1^H NOESY spectra of *s*Vpr^1-20 ^in H_2_O:D_2_O (9:1, v/v) at pH 7 prior to (red signals) and after addition of 50 μl (0.1 mg) CypA (green signals). Notice the appearance of exchange peaks originating from enhanced prolyl *cis/trans *interconversion rate after addition of CypA. **B_1_**: Expanded Hα-Hβ region of the 2D ^1^H-^1^H TOCSY spectrum of *s*Vpr^21-40 ^in H_2_O:D_2_O (9:1, v/v) at pH 7 showing the signals of *trans *and *cis *Pro-35. **B_2_**: Superimposed expanded Hα-Hβ regions of the 2D ^1^H-^1^H NOESY spectra of *s*Vpr^21-40 ^in H_2_O:D_2_O (9:1, v/v) at pH 7 prior to (red signals) and after addition of 100 μl (0.2 mg) CypA (green signals). Notice the appearance of exchange peaks originating from enhanced prolyl *cis/trans *interconversion rate after addition of CypA.

**Figure 3:**
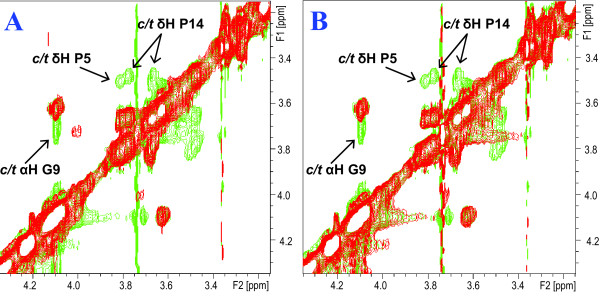
**Catalysis of isomerisation of Pro-5,10 and 14 of Vpr^1-20 ^by CypA**. **A**: Superimposed expanded Hα-Hβ region of the 2D ^1^H-^1^H NOESY spectra of *s*Vpr^1-20 ^in H_2_O:D_2_O (9:1, v/v) at pH 7 prior to (red signals) and after addition of 50 μl (0.1 mg) CypA (green signals). Notice the appearance of exchange peaks originating from enhanced prolyl *cis/trans *interconversion rate after addition of CypA. **B**: Superimposed expanded Hα-Hβ region of the 2D ^1^H-^1^H NOESY spectra of *s*Vpr^1-20 ^in H_2_O:D_2_O (9:1, v/v) at pH 7 after addition of 50 μl (0.1 mg) CypA (green signals) and after additional addition of 5 μl (0.1 mg) cyclosporine A (red signals). Notice that the prolyl *cis/trans *related exchange peaks detected after addition of CypA disappear after addition of cyclosporine A.

In an initial series of 2D NMR experiments (2D ^1^H TOCSY, ROESY and NOESY NMR spectra), the ^1^H chemical shifts of the all-*trans *isomers of *s*Vpr^1-20^, *s*Vpr^21-40 ^and *s*Vpr^25-40 ^in aqueous phosphate buffer solution at pH 7 containing 10% v/v D_2_O were completely assigned (Additional file 1, Table S1, Additional file 1, Table S2, Additional file 1, Table S3, Additional file 1, Table S4, Additional file 1, Table S5, Additional file 1, Table S6 and Additional file 1, Table S7). In accordance with previous investigations in aqueous organic solvent [19] the NMR spectra of *s*Vpr^1-20 ^revealed two sets of ^1^H NMR signals originating from the same residues in the sequence (Fig. 2). In each case one set of signals was more intense than the second set, and the corresponding amidic protons showed significant chemical shift differences. The fact that the residues showing the largest shift differences were those either adjacent to or very close to each of the Pro residues indicated that the weaker sets of signals in each case arose from the *cis *isomers of the Pro residues. The identification of sets of signals for Ala-4, Gly-9 and Tyr-15 (Fig. 2A), in addition to the ^1^H NMR signals originating from the *cis *isomers of Pro-5 and 14 (Fig. 3), is a strong indication that all Pro residues are involved in such processes under the conditions used. Similarly, ^1^H NMR signals originating from the *cis *isomer of Pro-35 were observed in the 2D ^1^H NMR spectra of *s*Vpr^21-40 ^(Fig. 2B) and *s*Vpr^25-40^.

It is particularly noteworthy that exchange peaks between analogous ^1^H signals of the *cis *and *trans *isomer were not observed in the NOESY spectra of any of the pure peptides, which is a clear indication that only a slow *cis/trans *interconversion on the NMR time scale is taking place in the absence of enzymatic catalysis. As a consequence of the relatively large energy barrier, uncatalyzed isomerisation is a rather slow process with an interconversion time on the order of several minutes [29,30].

### CypA catalyzes the prolyl *cis/trans *isomerisation of Pro-35 of sVpr^21-40 ^and sVpr^25-40^

NMR spectroscopy provides information about prolyl *cis/trans *isomerase interaction of CypA with Pro-containing peptides and proteins at atomic resolution. Originally, prolyl *cis/trans *isomerase activity of CypA with relatively short model peptides, containing one Pro residue, was revealed by line shape analysis of 1 D spectra [31-33]. Exchange spectroscopy including 2D ^1^H-^1^H NOESY and ROESY NMR experiments have been applied to determine prolyl *cis/trans *isomerase interactions of CypA with unlabelled model peptides [23,34,35]. Selective interaction of Pro-90 of HIV-1 Capsid was revealed by 3D ^15^N-edited NOESY-HSQC of ^15^N-labelled N-terminal (1-146) and full-length HIV-1 Capsid, in addition to a fragment of the HIV-1 Gag polyprotein containing the full Matrix protein and the N-terminal domain of Capsid [12,13].

**Figure 4:**
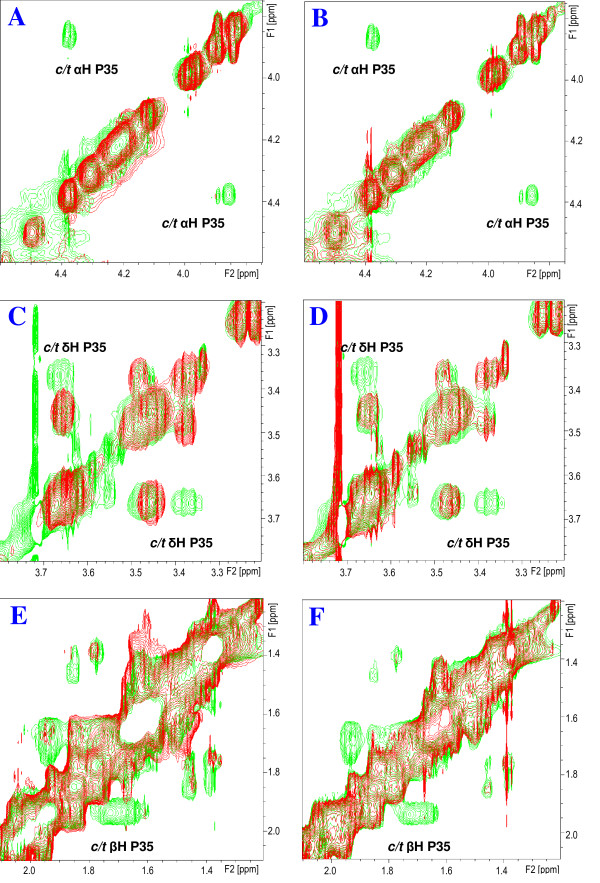
**Catalysis of isomerisation of Pro-35 of Vpr^21-40 ^by CypA**. **A**, **C**, **E**: Superimposed expanded Hα-Hβ regions of the 2D ^1^H-^1^H NOESY spectra of *s*Vpr^21-40 ^in H_2_O:D_2_O (9:1, v/v) at pH 7 prior to (red signals) and after addition of 100 μl (0.2 mg) CypA (green signals). Notice the appearance of exchange peaks originating from enhanced prolyl *cis/trans *interconversion rate after addition of CypA. **B**, **D**, **F**: Superimposed expanded Hα-Hβ region of the 2D ^1^H-^1^H NOESY spectra of *s*Vpr^21-40 ^in H_2_O:D_2_O (9:1, v/v) at pH 7 after addition of 100 μl (0.2 mg) CypA (green signals) and after additional addition of 5 μl (0.1 mg) cyclosporine A (red signals). Notice that the prolyl *cis/trans *related exchange peaks detected after addition of CypA disappear after addition of cyclosporine A.

When the complete series of NMR experiments allowing full assignment of the ^1^H chemical shifts of *s*Vpr^21-40 ^were recorded, CypA was added to the peptide solution at a molar ratio *s*Vpr^21-40^-CypA 140:1, and analogous NMR experiments were then recorded. In the presence of catalytic amounts of CypA, strong exchange peaks between related signals of the *cis *Pro-35 and *trans*-Pro-35 isomer of *s*Vpr^21-40 ^were observed (Fig. 4A, C and 4E). In particular, strong exchange peaks between *cis*-Hα/*trans*-Hα and *cis*-Hδ/*trans*-Hδ of Pro-35 were observed in the NOESY spectra after addition of catalytic amounts of CypA.

To confirm that CypA interacts with Pro-35 of *s*Vpr^21-40 ^and Pro-35 of *s*Vpr^25-40 ^as a prolyl *cis/trans *isomerase, an excess of cyclosporine A (CsA), a selective inhibitor of prolyl *cis/trans *isomerase activity of CypA was added, and a complete series of NMR spectra were again recorded. The NOESY and ROESY NMR spectra of *s*Vpr^21-40^, after the sequential addition of CypA and cyclosporine A, revealed that the prolyl *cis/trans *exchange crosspeaks disappeared after addition of cyclosporine A, and the NMR spectra closely resembled those of pure *s*Vpr^21-40 ^(Fig. 4B, D and 4F). Similar results were observed for *s*Vpr^25-40^. Thus, CypA behaves as a prolyl *cis/trans *isomerase, causing an increase in the interconversion rate of Pro-35 of Vpr.

### CypA catalyzes the prolyl *cis/trans *interconversion of Pro-5, -10 and -14 of sVpr^1-20^

The NMR spectra of *s*Vpr^1-20 ^in aqueous phosphate buffer at pH 7 revealed *cis/trans *proline isomerism of all Pro residues of this peptide through the observation of separate signals for the all-*trans *and various *cis *isomers originating from a slow equilibrium under non-catalytic conditions.

**Figures 5:**
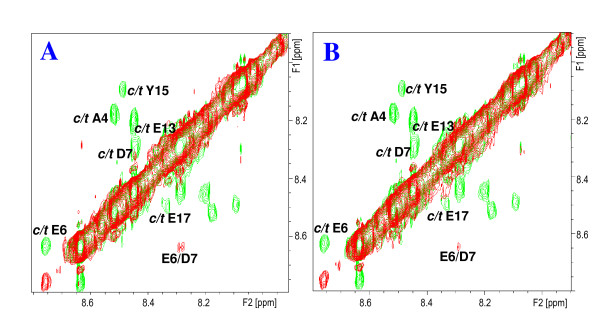
**Catalysis of isomerisation of Pro-5,10 and 14 of Vpr^1-20 ^by CypA**. **A**: Superimposed expanded HN-HN regions of the 2D ^1^H-^1^H NOESY spectra of *s*Vpr^1-20 ^in H_2_O:D_2_O (9:1, v/v) at pH 7 prior to (red signals) and after addition of 50 μl (0.1 mg) CypA (green signals). Notice the appearance of exchange peaks originating from enhanced prolyl *cis/trans *interconversion rate after addition of CypA. **B**: Superimposed expanded HN-HN regions of the 2D ^1^H-^1^H NOESY spectra of *s*Vpr^1-20 ^in H_2_O:D_2_O (9:1, v/v) at pH 7 after addition of 50 μl (0.1 mg) CypA (green signals) and after additional addition of 5 μl (0.1 mg) cyclosporine A (red signals). Notice that the prolyl *cis/trans *related exchange peaks detected after addition of CypA disappear after addition of cyclosporine A.

In order to reveal a potential interaction of CypA with the highly conserved Pro-5, -10 and -14 of Vpr, complete series of NMR experiments allowing full assignment of the ^1^H chemical shifts of *s*Vpr^1-20 ^were recorded, followed by addition of catalytic amounts of CypA (molar ratio *s*Vpr^1-20^-CypA 224:1; molar ratio *s*Vpr^1-20 ^proline substrate-CypA 672:1). After addition of catalytic amounts of CypA, particularly the HN-HN region of the 2D ^1^H-^1^H NOESY NMR spectrum of *s*Vpr^1-20 ^showed strong *cis/trans *exchange peaks between related protons of the all-*trans *isomer and various *cis *isomers originating from *cis *Pro-5, *cis *Pro-10 and *cis *Pro-14, respectively (Fig. 5A). This was further confirmed by the observation of exchange peaks for Hα of Gly-9 and those of Hδ of Pro-5 and Pro-14 (Fig. 3A). As described above for *s*Vpr^21-40 ^containing Pro-35, cyclosporine A was added and a further series of NMR spectra was recorded that showed the disappearance of the prolyl *cis/trans *exchange crosspeaks (Fig. 3B and 5B). Although Pro-5, -10 and -14 of Vpr are highly conserved [31], any interactions involving these residues have not previously been identified.

Thus we have direct experimental evidence that all four conserved Pro residues in Vpr undergo *cis/trans *isomerism in aqueous solution at pH 7 that is catalyzed by CypA. Only small amounts of enzyme are required and the NMR method is sufficiently sensitive to detect these effects in ratios of substrate to enzyme as high as 672:1. The applied relative proportions of enzyme to substrate were considerably lower than the relative proportions of CypA (enzyme to substrate 1:8-1:300) used in previous studies with NMR techniques [12,13,23,31-35].

### Determination of *cis/trans *interconversion rate by ROESY NMR experiments

To obtain the rate constant of the prolyl *cis/trans *interconversion, a series of ROESY experiments with variable mixing times were performed (Additional file 1, Table S8 and Additional file 1, Fig. S2). For each mixing time, volume integration of the well-resolved prolyl *cis/trans *exchange crosspeaks originating from *cis*HαPro-35 -*trans*HαPro-35 were performed, and based thereupon, the reaction rate constant was calculated. The mean reaction rate was calculated using the data from ROESY experiments with mixing times 200-500 ms. Mean reaction rate constant for the prolyl *cis/trans *interconversion was determined to be 0.19 ± 0.01 s^-1^

### Characterization of CypA-Vpr interaction using surface plasmon resonance spectroscopy

**Figure 6:**
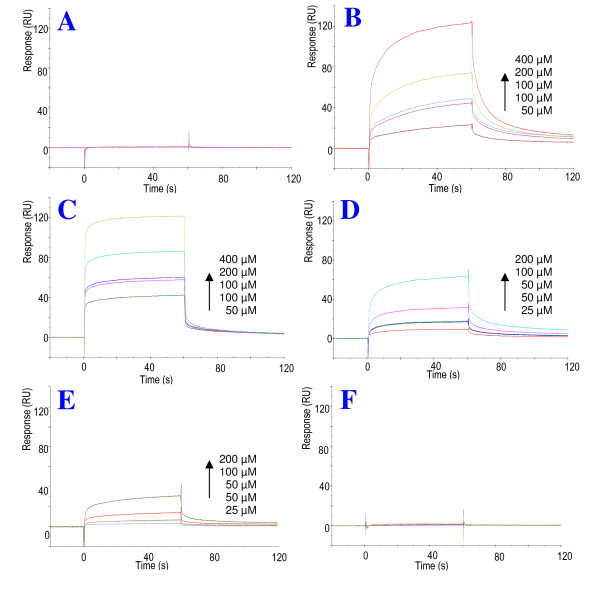
**Characterization of CypA binding to N-terminal peptides of *s*Vpr**. Synthetic Vpr^1-20^(**A**), Vpr^21-40 ^(**B**), Vpr^25-40 ^(**C**), Vpr^1-40^(**D**) and synthetic mutants carrying Pro to Asn exchanges at amino acid positions 5, 10 and 14(*s*Vpr^1-40^P5,10,14N) (**E**) and at position 35 (*s*Vpr^1-40 ^P35N) (**F**) were tested for binding to immobilized recombinant CypA using a SPR biosensor system. **A**, **B **and **C **were injected at concentrations ranging from 0-400 μM over CM5 chip immobilized with 918 RU CypA, while **D**, **E **and **F **were injected at concentrations ranging from 0-200 μM. The curves A, B, C, D and E were best fit to a two state reaction model (Fig. 7). k_a1_, k_a2_, k_d1_, k_d2 _and K_D _were calculated for respective sensograms (Table 1).

Previous Biacore studies [9] concluded that Pro-35 was a determinant for the ability of N-terminal Vpr peptides to interact with CypA. *s*Vpr^1-20 ^or any mutant N-terminal Vpr peptide lacking Pro-35 did not interact with CypA in the Biacore assay. Pure enzymatic interactions such as the observed catalysis of prolyl *cis/trans *isomerisation of N-terminal Vpr peptides by CypA will not be detected by SPR because of the short lifetime of the enzyme-substrate (ES) complexes (between 10^-7 ^and 10^-4 ^sec according to references [36] and [37]). We confirmed that the N-terminal peptides *s*Vpr^1-40^, *s*Vpr^21-40 ^and the mutant *s*Vpr^1-40 ^P5,10,14N bind to CypA, while *s*Vpr^1-20 ^and the mutant *s*Vpr^1-40 ^P35N failed to bind (Fig. 6). Thus, the binding region of N-terminal Vpr peptides for the observed interaction with CypA subsequently involves Pro-35.

**Figure 7:**
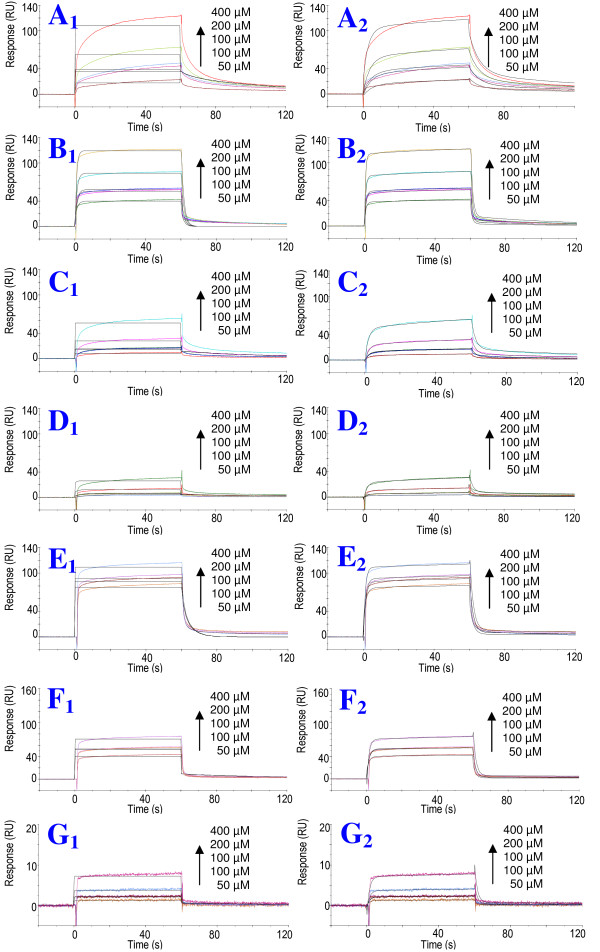
**Optimized fit of SPR sensograms to binding models**. The sensograms were fit to the binding models 1:1 (Langmuir) (**A_1_-G_1_**) and two-state reaction (conformational change) model (**A_2_-G_2_**). Black lines show the best curve fit in each instance. The curves originating from the interactions of *s*Vpr^21-40 ^(**A**), *s*Vpr^25-40 ^(**B**), *s*Vpr^1-40^(**C**), *s*Vpr^1-40 ^P5,10,14N (**D**), *s*Vpr^30-40 ^(**E**), *s*Vpr^32-38 ^(**F**) and *s*Vpr^33-37^(**G**) with CypA were considered to fit to two state reaction model (sensogram **A_2_-G_2_**) best.

Kinetic analysis of the Biacore binding data showed deviations from a pseudo-first order 1:1 binding model. The binding curves of CypA with *s*Vpr^25-40^, *s*Vpr^21-40^, *s*Vpr^1-40 ^and the short peptides *s*Vpr^30-40 ^and *s*Vpr^32-38 ^were best described by a two-state binding interaction model (Fig. 7, Additional file 1, Fig. S3). The minor deviation observed between the experimental and modelled curve for *s*Vpr^21-40 ^at the highest concentration, i.e. 400 μM, may be due to aggregation of the peptide at this concentration. The two-state binding model, also called the conformational change model, is based on a 1:1 binding of an analyte to the immobilized ligand followed by a conformational change in the complex. This model should be regarded as indicative, rather than as direct evidence, for a conformational change upon binding [38]. However, our NMR data confirm that CypA acts as a prolyl *cis/trans *isomerase of the highly conserved Pro residues of N-terminal Vpr including Pro-35, hence a conformational change upon binding is probable. Thus, CypA catalyzes the prolyl *cis/trans *interconversion during binding to the *s*Vpr peptides. The interconversion rate constant derived from the ROESY NMR experiments was determined to be 0.19 ± 0.01 s^-1^. Thus, a peptide-CypA complex would undergo approximately 12 prolyl *cis/trans *interconversions during the 60 sec. association phase of the Biacore experiment. From the two-state binding model the association rate (k_a1_), dissociation rate (k_d1_), forward rate (k_a2_) and backward rate (k_d2_) constants were calculated, and subsequently the magnitude of the dissociation constants (K_D_) could be calculated (1/((k_a1_/k_d1_)*(k_a2_/k_d2_)) (Table 1).

**Table 1:** Estimated kinetic constants for binding of HIV-1 N-terminal Vpr peptides to CypA.

Peptide	Amino acid sequence	Kinetic constants by two state reaction model		
		K_D _(M)	ka_1 _(1/Ms) ka_2 _(1/s)	kd(1/s)
*s*Vpr^21-40^	ELLEELKSEAVRHF**P^35 ^**RIWLH	**2.01 × 10^-4^**	k_a1 _= 466 k_a2 _= 0.0084	k_d1 _= 0.164 k_d2 _= 0.011
*s*Vpr^25-40^	ELKSEAVRHF**P^35 ^**RIWLH	**4.04 × 10^-4^**	k_a1 _= 2004 K_a2 _= 0.0028	k_d1 _= 0.9415 k_d2 _= 0.0173
*s*Vpr^1-40^	MEQA**P^5^**EDQG**P^10^**QRE**P^14^**YNEWTTLELLEELKSEAVRHF**P^35 ^**RIWLH	**2.84 × 10^-4^**	k_a1 _= 752 k_a2 _= 0.0073	k_d1 _= 0.37 k_d2 _= 0.0099
*s*Vpr^1-40 ^P5,10,14 N	MEQA**N^5^**EDQG**N^10^**QRE**N^14^**YNEWT TLELLEELKSEAVRHF**P^35^** RIWLH	0.00615*	k_a1 _= 46* k_a2 _= 0.0047	k_d1 _= 0.56* k_d2 _= 0.0048
*s*Vpr^30-40^	AVRHF**P^35 ^**RIWLH	**1.67 × 10^-4^**	k_a1 _= 2467 k_a2 _= 0.00145	k_d1 _= 0.527 k_d2 _= 0.0053
*s*Vpr^32-38^	RHF**P^35 ^**RIW	**4.87 × 10^-4^**	k_a1 _= 924 k_a2 _= 0.0011	k_d1 _= 0.67 k_d2 _= 0.0023
*s*Vpr^33-37^	HF**P^35^**RI	**0.00357**	k_a1 _= 120.4 k_a2 _= 0.00138	k_d1 _= 0.66 k_d2 _= 0.0025

*ka_1 _beyond the values measurable by the Biacore instrument.

The calculated dissociation constants were all of the same order of magnitude for the N-terminal Vpr peptides *s*Vpr^1-40^, *s*Vpr^21-40^, *s*Vpr^25-40^, *s*Vpr^30-40 ^and *s*Vpr^32-38 ^containing Pro-35 (Table 1). Reliable dissociation constants for *s*Vpr^1-40 ^P5,10,14N, which also contains Pro-35 and binds to CypA (Fig. 6E), could not be determined with high confidence. This may be due to relatively poor solubility of the latter peptide in aqueous buffer solution at pH 7.

The two facts, that *s*Vpr^1-20 ^did not bind to CypA in the Biacore assay (Fig. 6) although CypA was shown by our NMR studies to interact with these Pro residues of the peptide as a prolyl *cis*/*trans *isomerase (Fig. 3, 5) and that the presence of Pro-35 on the other hand is required for binding, indicate the observed specific binding of N-terminal Vpr peptides to CypA require a binding region that is structurally dependent on Pro-35.

**Figure 8:**
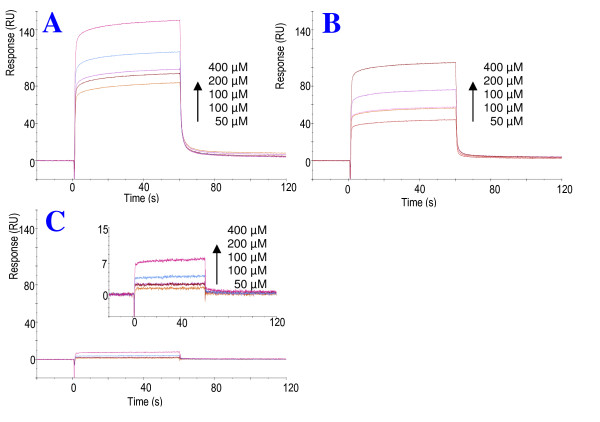
**Characterization of CypA binding region of the N-terminus of Vpr**. Synthetic Vpr^30-40 ^(**A**), Vpr^32-38 ^(**B**), Vpr^33-37^(**C, D**) were tested for binding to immobilized recombinant CypA using SPR biosensor system. Individual peptides were injected at concentration ranging from 0-400 μM over CM5 chip immobilized with 980 RU CypA. The curves were best fit to a two state model, and k_a1_, k_a2_, k_d1_, k_d2 _and K_D _were calculated for the respective sensograms (Table 1.).

To identify and determine the specific binding region of N-terminal Vpr to CypA, SPR studies of the shorter Vpr peptides *s*Vpr^30-40^, *s*Vpr^32-38 ^and *s*Vpr^33-37 ^were performed. The sensograms revealed that *s*Vpr^30-40^and *s*Vpr^32-38 ^maintain the strong binding to CypA similar to that of longer N-terminal Vpr peptides (Fig. 8). In contrast, the shortest peptide *s*Vpr^33-37 ^binds considerably weaker. The remarkably weaker response of the binding curves of *s*Vpr^33-37 ^to CypA compared with the longer peptides, demonstrates that the shortest peptide sequence maintaining strong binding is the heptapeptide *s*Vpr^32-38^. Thus the seven-residue motif RHFPRIW centred at Pro-35 defines the region for strong specific binding to CypA. In keeping with our findings, Zander et al. [9] and Ardon et al. [10] reported that CypA co-immunoprecipitates with wild-type Vpr, while mutation of Pro-35 caused loss of this phenomenon.

## Discussion

As a continuation of our previous discovery of the interaction of CypA with Vpr, these interactions have in this work been characterized in detail at atomic resolution. Direct experimental evidence that all four conserved Pro residues in Vpr undergo *cis/trans *isomerism in aqueous solution at pH 7 that is catalyzed by CypA was achieved. Only small amounts of enzyme are required and the NMR method is sufficiently sensitive to detect these effects in ratios of substrate to enzyme as high as 672:1.

The apparent differentiation between the results originating from interaction studies performed by NMR spectroscopy and SPR indicates a different additional mode of interaction observed in the latter case. The fact that only N-terminal Vpr peptides containing Pro-35 bind to CypA in the Biacore assay and strong binding is maintained in the heptapeptide *s*Vpr^32-38^, although CypA also catalyzes prolyl *cis/trans *interconversions of Pro-5, 10 and 14 of *s*Vpr^1-20 ^as shown by NMR spectroscopy at atomic resolution, is convincing evidence that CypA only binds specifically to peptides containing the seven-residue motif RHFPRIW centred at Pro-35 of N-terminal Vpr. With the exception of Ile-37, residues comprising the binding region of N-terminal Vpr to CypA are highly conserved [26]. This region includes the loop region connecting helices 1 and 2 of Vpr, in addition to Arg-32 and His-33 that terminates the well-defined helix 1 of Vpr.

The situation found here is analogous to the NMR study reported by Bosco et al. [12] where CypA not only interacts as a chaperone with Pro-90 of N-terminal HIV-1 Capsid but also selectively catalyzes the prolyl *cis/trans *interconversion of the Gly-89 -Pro-90 bond. Crystal structure of the complex of CypA with the N-terminal HIV-1 Capsid peptide (residues 1-151) revealed that the binding region of HIV-1 Capsid to CypA encompasses the nine residues 85 to 93 (PVHAGPIAP) belonging to a loop region connecting helices 4 and 5, respectively, of HIV-1 Capsid [39]. Based on X-ray crystallography, Vajdos et al. [40] reported that the HIV-1 Capsid-derived hexapeptide HAGP^90^IA maintained the strong interaction with CypA, thus limiting the binding region of HIV-1 Capsid to include these six residues.

Piotukh et al. [41] used phage display to delineate the requirements for stable binding of CypA to linear peptide motifs containing the consensus motif FGPXLp that is present in many human proteins. This binding motif is quite different from the sequence RHFPRIW found here that defines the binding region of N-terminal Vpr to CypA and also differs from the binding region of HIV-1 Capsid (HAGPIA), indicating that the Pro-dependent binding pocket of CypA is tolerant of substrates with several different sequences.

Pro-35 in Vpr is located in a loop region separating helix 1 and helix 2 [25]. Structural data for an N-terminal mutant of Vpr in which Pro-35 is exchanged for Asn indicate a merge of helices 1 and 2 leading to a significant conformational change in the hydrophobic core of Vpr [11]. This will thus disrupt the hydrophobic interactions of helices 1 and 2 with helix 3 that were observed by long-range NOEs [25]. The tertiary structure of *wt *Vpr consists of three well-defined α-helices consisting of residues 17-33 (helix 1), 38-50 (helix 2) and 56-77 (helix 3), respectively, folded around a hydrophobic core constituted of Leu, Ile, Val and aromatic residues [25]. According to Morellet et al. [25], the hydrophilic accessible domains of Vpr exposed to the solvent molecules comprise the acidic residues Glu-21, Glu-22, Glu-24, Glu-25, Asn-28 and Glu-29 of helix 1, the basic residues Arg-62, Arg-73, Arg-77, Gln-65 and Gln-66 of helix 3 and the basic residues Arg-80, Arg-87, Arg-88, Arg-90 and Lys-95 of the flexible C-terminal domain, respectively. The fact that the mutant Vpr R80A failed to co-immunoprecipitate with CypA [10] may indicate that the binding of full-length Vpr to CypA is also dependent on the maintenance of the tertiary structure of Vpr. Clearly there are at least two highly conserved residues in the molecule that are crucial for maintaining the interaction of CypA with Vpr, namely Pro-35 and Arg-80.

Previous literature has indicated that CypA is relatively tolerant as a prolyl *cis/trans *isomerase to a variety of different substrates [21]; however, these studies have been performed exclusively on short peptides comprised of four amino acids and containing only one Pro residue. To our knowledge catalysis by CypA of prolyl *cis/trans *interconversions of more than one Pro residue in a peptide containing several Pro residues has not previously been reported. However, exclusively selective prolyl *cis/trans *isomerase catalysis mediated by CypA on only one Pro unit have been reported for peptides containing more than one Pro residue [12,13,23].

The binding curves from Biacore analyses of the specific interaction of Pro-35 were consistent with a two-state binding interaction model involving a conformational change during the binding. This is in accord with the catalysis of prolyl *cis/trans *isomerism observed by NMR in solution, indicating that the RHFPRIW motif of N-terminal Vpr binds the catalytic site of CypA, which maintains the ability to catalyze the prolyl *cis/trans *interconversion during binding.

The fact that CypA specifically binds to the inter-helical loop region included in the RHFPRIW binding motif centred at Pro-35, under solution conditions where Vpr has a relatively random structure, suggests a mechanism in which CypA may act as a folding chaperone. Whether this plays a role in the *de novo *synthesis of Vpr, in which CypA appears to be involved, remains to be determined [9]. Clearly the functional consequences of the interactions of CypA still remain to be determined. Further studies will necessitate the evaluation of these interactions in the context of the full length Vpr molecule in the various environments that it encounters *in vivo*.

## Conclusions

Binding of CypA to a defined inter-helical loop region of HIV-1 Vpr is highly specific, which is analogous to the previously characterised HIV-1 Capsid-CypA interaction. The loop region centred at Pro-35 of Vpr comprising the binding motif to CypA maintains the integrity of the N-terminal and middle helices of Vpr and is functionally important for incorporation of Vpr into virus particles and replication of R5-tropic HIV-1 in human lymphoid tissue [28]. CypA acts as a catalyst of prolyl *cis*/*trans *interconversions of Pro 5, 10, 14 and 35 of N-terminal HIV-1 Vpr, which may indicate a folding chaperone role of CypA in HIV replication compatible with the fact that inhibitors of CypA, which inhibit CypA-Vpr binding [9,10] and CypA-mediated prolyl *cis*/*trans *catalysis, also suppress HIV-1 replication in cell culture.

## Methods

### Peptide synthesis

The synthesis, purification and molecular characterization of synthetic Vpr (*s*Vpr) fragments, *s*Vpr^1-40^, *s*Vpr^1-40 ^P35N, *s*Vpr^1-40 ^P5,10,14N, *s*Vpr^1-20^, *s*Vpr^21-40^, *s*Vpr^25-40^, *s*Vpr^30-40^, *s*Vpr^32-38 ^and *s*Vpr^33-37 ^were performed as described in detail elsewhere [7,19] and all showed the expected high quality HPLC peaks and correct molecular masses by MALDI-MS and positive ion ESI-MS.

### Cyclosporine A and CypA

Cyclosporine A was purchased from Sigma (C3662; CAS number 59865-13-3). The procedure on which the purification of recombinant human CypA is based has previously been described by others [42]. The *E. coli *strain BL21(DE3) (Invitrogen) was transformed with an pET11c expression vector encoding human CypA [43]. A 4-liter culture of *E. coli *BL21(DE3) was grown at 37°C in a LB medium containing ampicillin to an A_595 _of 0.8 and isopropyl-β-D-thiogalactopyranoside (IPTG) was added to a final concentration of 1 mM. The incubation was continued for additional 8 h. The cells were harvested (3000 g, 15 min), washed with Tris buffer (1 mM DTT, 20 mM TrisHCl, pH 7, 8) and 12 g cell pellet was resuspended in 150 ml of the same buffer. Lysis was performed on ice using a BL12 sonifier (Branson). After separation of the cell debris by centrifugation (48000 g, 15 min) nucleic acids were precipitated by protamine sulphate (0.4% final concentration). The crude cell extract was dialyzed against the Tris buffer overnight and loaded onto a DEAE-Sepharose column (HiPrep16/10, GE Healthcare) equilibrated with 4 column volumes (CV) of Tris-buffer and the flow-through fractions were collected, containing essentially pure CypA. These fractions were combined and dialyzed against a 30 mM MES buffer pH 6.0 overnight. For final purification and concentration of CypA, the dialyzed solution was loaded on a MonoS cation exchange column (5/50 GL, GE Healthcare) which was equilibrated with 4 CV of the MES buffer. The final elution step of the bound CypA was performed with a 0-1 M NaCl gradient and yielded 30 mg of highly concentrated and pure CypA fractions, which were analysed by Coomassie staining and Maldi MS to check the purity and the correct molecular weight of the protein (Additional file 1, Fig. S4).

### Mass Spectrometry

Matrix assisted laser desorption ionization mass spectra (MALDI-MS) were recorded on a Voyager-DE PRO BioSpectrometry Workstation from Applied Biosystems. Samples were dissolved in 50% aqueous acetonitrile and *α*-cyano-4-hydroxycinnamic acid was used as matrix. Positive ion electrospray ionization mass spectra (ESI-MS) were recorded on a micromass Q-Tof-2 mass spectrometer. Samples were dissolved in 70% aqueous methanol and infused into the electrospray chamber with a needle voltage of 0.9 kV at a flow rate of 40 nl/min.

### Nuclear Magnetic Resonance (NMR) Spectroscopy

2D ^1^H Total correlation spectroscopy (TOCSY), Nuclear Overhauser enhancement spectroscopy (NOESY) and Rotating frame Overhauser enhancement spectroscopy (ROESY) NMR experiments were performed at 600.13 MHz on a Bruker Avance 600 MHz instrument equipped with an UltraShield Plus magnet and a triple resonance cryoprobe with gradient unit. Individual samples were dissolved in 600 μl 50 mM aqueous phosphate buffer pH 7.0 containing 10% D_2_O (v/v), at concentrations between 1-2 mM. The 2D NMR experiments were performed at 300 K without spinning with mixing times of 110 ms for the TOCSY experiments, 250 ms for the NOESY experiments and 500 ms for the ROESY experiments, respectively. Efficient suppression of the water signal was achieved with application of excitation sculpting in the 1 D ^1^H and the 2D ^1^H TOCSY and NOESY NMR experiments [44] and *presat *in the 2D ^1^H ROESY experiments [45]. ^ 1^H signal assignments of the NMR spectra were achieved by identification of the individual spin systems in the 2D ^1^H TOCSY spectra, combined with observations of sequence-specific short-distance crosspeaks (Hα-HN i, i+1) in the 2D ^1^H-^1^H NOESY spectra [9,46]. Readily recognisable spin systems were used as starting points for correlation of the individual spin systems observed in the TOCSY and NOESY spectra with individual residues in the peptide sequences. Acquisition of data, processing and spectral analysis were performed with Bruker Topspin 1.3 software. Assigned NMR data of the N-terminal Vpr peptides have been deposited at the BMRB (Accession number 17003).

### Interactions of CypA with sVpr^1-20 ^and sVpr^21-40 ^peptides

After acquisition of 1 D ^1^H and 2D ^1^H TOCSY, NOESY and ROESY NMR spectra of pure *s*Vpr^1-20 ^and *s*Vpr^21-40^, 50-100 μl 0.11 mM CypA dissolved in aqueous Tris buffer at pH 7 was added to the individual peptide solutions, followed by acquisition of identical series of NMR spectra (1 D ^1^H and 2D ^1^H TOCSY, NOESY and ROESY) to those of the pure peptides. Exchange peaks occurring in the spectra after addition of CypA were identified by supposition of analogous NOESY and/or ROESY spectra prior to and after addition of CypA using Bruker Topspin 1.3 software.

### Determination of rate constant for Pro cis/trans interconversion by ROESY NMR

A series of ROESY experiments of Vpr^32-38 ^(sample concentration 4.2 mM) with addition of 50 μl 0.11 mM CypA (sample concentration of CypA 8.5 μM; total volume 650 μl); relative proportions of substrate to enzyme 494:1, maintaining the same experimental condition as described above but with variable mixing time (500 ms, 450 ms, 400 ms, 350 ms, 300 ms, 250 ms 200 ms 150 ms, 100 ms and 50 ms, respectively) were performed. Volume integration of exchange peaks were carried out with Sparky software [47] and rate constants for each mixing time were calculated as described in Keller et al. [48].

### Specific inhibition of prolyl cis/trans isomerase interactions of CypA with sVpr^1-20 ^and sVpr^21-40 ^by addition of cyclosporine A

The catalytic prolyl *cis/trans *isomerase interactions of CypA with *s*Vpr^1-20 ^and *s*Vpr^21-40 ^was inhibited by addition of excessive amounts of cyclosporine A dissolved in 5 μl deuterated dimethylsulfoxide (DMSO-*d6*). Addition of 6 μl pure DMSO did not inhibit the catalytic prolyl *cis/trans *isomerase interactions of CypA with a model peptide showing that CypA keeps its enzyme activity in 1% DMSO solution. The disappearance of NMR exchange peaks, originating from the catalytic prolyl *cis/trans *isomerase interactions of CypA with *s*Vpr^1-20 ^and *s*Vpr^21-40^, after addition of cyclosporine A was revealed by supposition of analogous NOESY and ROESY spectra prior to and after addition of cyclosporine A, using Bruker Topspin 1.3 software.

### Biacore Spectroscopy

SPR measurements were performed at 25°C on a Biacore T100 instrument (Biacore AB, Uppsala, Sweden) equipped with a CM5 research-grade sensor chip. CypA was immobilized in FC 2 and FC 4 to 180 and 918 RU (response units) respectively, using standard amine-coupling chemistry in two flow cells. FC 1 and FC 3 were treated according to FC 2 and FC 4 except for CypA immobilisation and functioned as reference cells. The synthetic Vpr peptides *s*Vpr^1-20^, *s*Vpr^21-40^, *s*Vpr^25-40^, *s*Vpr^30-40^, *s*Vpr^32-38 ^and *s*Vpr^33-37 ^were dissolved at four concentrations ranging from 50 to 400 μM in the running buffer (HBS-P buffer pH 7.4). Due to restricted solubility *s*Vpr^1-40^, *s*Vpr^1-40 ^P35N and *s*Vpr^1-40 ^P5,10,14N were dissolved at lower concentrations ranging from 25-200 μM in the same buffer. The samples were injected over the flow cells at a flow rate of 30 μl/min. Data were collected at 2.5 Hz during the 60 s association and 120 s dissociation phase, and were automatically corrected for bulk buffer effects and unspecific binding of Vpr peptides to the chip matrix.

### Analysis of biosensor data

Affinity, association and dissociation rate constants were obtained from sensograms by the Biacore T100 evaluation software version 2.0.1 in accordance with the global curve fit model. Sensorgram data for the four different concentrations were fitted to several binding models including 1:1 (Langmuir) binding model (A+B ↔ AB), two-state reaction (conformational change) model (A+B ↔ AB ↔ AB*) and heterogeneous ligand model (interaction one: A+B1 ↔ AB1; interaction two: A+B2 ↔ AB2). Kinetic constants were calculated for the best fitted model.

## Authors' contributions

All authors read and approved the final manuscript. SMS, TRR, US, VW and TF participated in planning the experimental work. SMS, TRR, VW and TF planned and performed the structural and functional NMR studies. RR, PH and PH synthesized the peptides. DM produced CypA. SPR measurements were performed by SMS, OH, AJR, KB and TF. VW, SMS and TF wrote the manuscript.

## Supplementary Material

Additional file 1 **Tables of ^1^H Chemical shifts of N-terminal Vpr peptides**. MALDI-TOF Mass spectrum of CypA. Chemical shift differences of the Hα-protons between the experimental values and those for random coil residues, for *s*Vpr^1-20 ^and *s*Vpr^21-40^. Calculated reaction rate constants for the prolyl *cis*/*trans *interconversion of Pro-35 of *s*Vpr^32-38^. Optimised fit residuals of SPR sensograms. Peak intensities of *cis/trans *crosspeaks versus diagonal signal intensities influenced by mixing times observed in the ROESY spectra of *s*Vpr^32-38^. Click here for file
