# Supplementary material for: The intriguing Cyclophilin A-HIV-1 Vpr interaction: prolyl cis/trans isomerisation catalysis and specific binding
Source: BMC Struct Biol. 2010 Oct 4;10:31. doi: 10.1186/1472-6807-10-31 (PMC2959089; doi:10.1186/1472-6807-10-31)
Supplement: Additional file 1 — Tables of 1H Chemical shifts of N-terminal Vpr peptides. MALDI-TOF Mass spectrum of CypA. Chemical shift differences of the Hα-protons between the experimental values and those for random coil residues, for sVpr1-20 and sVpr21-40. Calculated reaction rate constants for the prolyl cis/trans interconversion of Pro-35 of sVpr32-38. Optimised fit residuals of SPR sensograms. Peak intensities of cis/trans crosspeaks versus diagonal signal intensities influenced by mixing times observed in the ROESY spectra of sVpr32-38. [file 1472-6807-10-31-S1.DOC]

**The intriguing Cyclophilin A-HIV-1 Vpr interaction: prolyl *cis*/*trans* isomerisation catalysis and specific binding**

**Sara M Solbak^1,2^, Tove R Reksten^1,2,3^, Victor Wray^3^, Karsten Bruns^3^, Ole Horvli^4^, Arnt J Raae^4^, Petra Henklein^5^, Peter Henklein^5^, Rene Röder^5^, David Mitzner^6^, Ulrich Schubert ^6^ and Torgils Fossen ^1,2§^**

^1^ From the Department of Chemistry, University of Bergen, N-5007 Bergen, Norway

^2^ Centre of Pharmacy, University of Bergen, N-5007 Bergen, Norway

^3^ Department of Structural Biology, Helmholtz Centre for Infection Research, D-38124 Braunschweig, Germany

^4^ Department of Molecular Biology, University of Bergen, N-5020 Bergen, Norway

^5^ Institute of Biochemistry, Charité Universitätsmedizin-Berlin, D-10117 Berlin, Germany

^6^ Institute of Virology, University of Erlangen-Nürnberg, D-91054 Erlangen, Germany

^§^Corresponding author

**Table S1.**

^1^H chemical shifts of all-*trans* proline conformers of *s*Vpr^1-20^ at 300K in aqueous phosphate buffer:D_2_O (9:1, v/v) at pH 7

| Amino acid | NH | Hα | Hβ | Hγ | Hδ | Hε | NH/NH_2_ | Ar-H |
| --- | --- | --- | --- | --- | --- | --- | --- | --- |
| Met-1 |  | 4.43 |  |  |  |  |  |  |
| Glu-2 | 8.56 | 4.26 | 2.05  1.96 | 2.27 |  |  |  |  |
| Gln-3 | 8.51 | 4.34 | 2.09  1.97 | 2.37 |  |  | 6.82  7.57 |  |
| Ala-4 | 8.52 | 4.61 | 1.39 |  |  |  |  |  |
| Pro-5 |  | 4.41 | 2.33 | 2.05  1.91 | 3.82  3.68 |  |  |  |
| Glu-6 | 8.64 | 4.24 | 2.05  1.95 | 2.29 |  |  |  |  |
| Asp-7 | 8.29 | 4.61 | 2.68  2.73 |  |  |  |  |  |
| Gln-8 | 8.26 | 4.37 | 2.21  1.97 | 2.38 |  |  | 6.83  7.51 |  |
| Gly-9 | 8.30 | 4.10 |  |  |  |  |  |  |
| Pro-10 |  | 4.43 | 2.29 | 2.02  1.91 | 3.63 |  |  |  |
| Gln-11 | 8.48 | 4.31 | 1.99  2.10 | 2.38  2.21 |  |  | 6.83  7.51 |  |
| Arg-12 | 8.34 | 4.34 | 1.75  1.84 | 1.62 | 3.18 |  |  |  |
| Glu-13 | 8.45 | 4.56 | 1.87  2.00 | 2.30 |  |  |  |  |
| Pro-14 |  | 4.36 | 2.22  1.98 | 1.83 | 3.78  3.66 |  |  |  |
| Tyr-15 | 8.09 | 4.50 | 2.99 |  |  |  |  | 2/6 7.09  3/5 6.81 |
| Asn-16 | 8.12 | 4.58 | 2.60 |  |  |  | 7.50  6.84 |  |
| Glu-17 | 8.32 | 4.11 | 1.90  1.83 | 2.04 |  |  |  |  |
| Trp-18 | 8.07 | 4.71 | 3.28  3.36 |  |  |  |  | NH 10.10  2H 7.26  4H 7.63  5H 7.15  6H 7.25  7H 7.49 |
| Thr-19 | 7.81 | 4.21 | 4.13 | 1.08 |  |  |  |  |
| Leu-20 | 7.89 | 4.23 | 1.60 |  | 0.92  0.87 |  |  |  |

**Table S2.**

^1^H chemical shifts for *cis* proline conformers of *s*Vpr^1-20^ at 300K in aqueous phosphate buffer:D_2_O (9:1, v/v) at pH 7

|  | NH | Hα | Hβ | Hγ | Hδ | Hε | NH/NH_2_ | Ar-H |
| --- | --- | --- | --- | --- | --- | --- | --- | --- |
| Gln-3 | 8.67 |  |  |  |  |  |  |  |
| Ala-4 | 8.45 | 4.30 | 1.38 |  |  |  |  |  |
| Ala-4 | 8.18 | 4.44 | 1.32 |  |  |  |  |  |
| Pro-5 |  |  | 2.23 | 1.98  1.88 | 3.50  3.60 |  |  |  |
| Glu-6 | 8.76 | 4.25 | 1.96  2.07 | 2.3 |  |  |  |  |
| Asp-7 | 8.44 |  | 2.67 |  |  |  |  |  |
| Gln-8 |  | 4.43 |  |  |  |  |  |  |
| Gly-9 | 8.28 | 3.73 |  |  |  |  |  |  |
| Pro-10 |  | 4.62 | 2.40  2.11 | 1.82  1.94 | 3.55  3.58 |  |  |  |
| Gln-11 | 8.64 | 4.35 | 2.13 | 2.38 |  |  |  |  |
| Arg-12 | 8.51 | 4.37 | 1.84  1.74 | 1.63 | 3.17 |  |  |  |
| Arg-12 | 8.22 | 4.37 | 1.73  1.82 | 1.61 | 3.16 |  |  |  |
| Glu-13 | 8.60 | 4.25 | 1.94  2.06 | 2.30 |  |  |  |  |
| Glu-13 | 8.45 | 4.16 | 1.82  1.92 | 2.12  2.22 |  |  |  |  |
| Pro-14 |  | 4.66 | 2.30  2.09 | 1.90  1.72 | 3.48 |  |  |  |
| Tyr-15 | 8.49 | 4.53 | 3.01  3.05 |  |  |  |  | 2/6 7.09  3/5 6.77 |
| Asn-16 | 8.26 | 4.56 | 2.62 |  |  |  |  |  |
| Glu-17 |  |  |  |  |  |  |  |  |
| Trp-18 | 8.03 |  | 3.23  3.35 |  |  |  |  | NH 10.07  2H 7.24 |
| Thr-19 | 7.82 | 4.21 | 4.13 | 1.08 |  |  |  |  |
| Leu-20 | 7.71 | 4.15 | 1.58 |  | 0.90 |  |  |  |

**Table S3.**

1H chemical shifts for all-*trans* proline conformers of *s*Vpr21-40 at 300K in aqueous phosphate buffer:D2O (9:1, v/v) at pH 7

|  | HN | Hα | Hβ | Hγ | Hδ | Hε | NH/NH_2_ | Ar-H |
| --- | --- | --- | --- | --- | --- | --- | --- | --- |
| Glu-21 |  | 4.38 | 1.93 | 2.24 |  |  |  |  |
| Leu-22 | 8.43 | 4.32 | 1.62 | 1.62 | 0.88 |  |  |  |
| Leu-23 | 8.19 | 4.31 | 1.69 |  | 0.88 |  |  |  |
| Glu-24 | 8.38 | 4.20 | 1.97 | 2.23 |  |  |  |  |
| Glu-25 | 8.41 | 4.21 | 1.98 | 2.23 |  |  |  |  |
| Leu-26 | 8.05 | 4.12 | 1.72 |  | 1.05  0.77 |  |  |  |
| Lys-27 | 8.23 | 4.30 | 1.80 | 1.43 | 1.68 | 2.96 | 7.22 |  |
| Ser-28 | 8.21 | 4.40 | 3.87 |  |  |  |  |  |
| Glu-29 | 8.44 | 4.23 | 2.08  1.95 | 2.28 |  |  |  |  |
| Ala-30 | 8.15 | 4.25 | 1.38 |  |  |  |  |  |
| Val-31 | 7.85 | 3.97 | 2.02 | 0.87 |  |  |  |  |
| Arg-32 | 8.11 | 4.20 | 1.64 | 1.47 | 3.11 |  | 6.98 |  |
| His-33 | 8.15 | 4.60 | 3.11  3.00 |  |  |  |  | 2H 8.02  4H 7.03 |
| Phe-34 | 8.17 | 4.81 | 3.11  2.89 |  |  |  |  | 2/6 7.23  3/5 7,31  4 7,27 |
| Pro-35 |  | 4.38 | 2.24 | 1.92  1.83 | 3.66  3.46 |  |  |  |
| Arg-36 | 8.35 | 4.18 | 1.66 | 1.52 | 3.08 |  | 6.98 |  |
| Ile-37 | 8.00 | 4.12 | 1.78 | 1.13  1.36 | 0.80 |  |  |  |
| Trp-38 | 8.23 | 4.76 | 3.13  3.23 |  |  |  |  | NH 10.13  2H 7.15  4H 7.60  5H 7.10  6H 7.20  7H 7.45 |
| Leu-39 | 8.02 | 4.24 | 1.41 |  | 0.81 |  |  |  |
| His-40 | 7.93 | 4.50 | 2.94  3.10 |  |  |  |  | 2H 7.99  4H 6.98 |

**Table S4.**

1H chemical for amino acids surrounding *cis* proline 35 of *s*Vpr21-40 aqueous phosphate buffer:D2O (9:1, v/v) at pH 7.

|  | HN | Hα | Hβ | Hγ | Hδ | Hε | NH/NH_2_ | Ar-H |
| --- | --- | --- | --- | --- | --- | --- | --- | --- |
| Val-31 | 7.82 | 4.00 | 2.03 | 0.87 |  |  |  |  |
| Arg-32 | 8.14 | 4.25 | 1.70 | 1.53 | 3.11 |  |  |  |
| His-33 |  |  |  |  |  |  |  |  |
| Phe-34 | 8.05 | 4.50 | 2.96 |  |  |  |  | 2/6 7.22 |
| Pro-35 |  | 3.85 | 1.85 | 1.69 | 3.48  3.38 |  |  |  |
| Arg-36 | 8.47 | 4.14 | 1.67 | 1.49 | 3.07 |  |  |  |
| Ile-37 |  |  |  |  |  |  |  |  |
| Trp-38 | 8.30 | 4.71 | 3.22  3.11 |  |  |  |  | NH 10.10  2H 7.17 |

**Table S5.**

^1^H chemical shifts for all-*trans* proline conformers of *s*Vpr^25-40^ at 300K in aqueous phosphate buffer:D_2_O (9:1, v/v) at pH 7

|  | NH | Hα | Hβ | Hγ | Hδ | Hε | NH/NH_2_ | Ar-H |
| --- | --- | --- | --- | --- | --- | --- | --- | --- |
| Glu-25 | - |  |  |  |  |  |  |  |
| Leu-26 |  |  |  |  |  |  |  |  |
| Lys-27 | 8.53 | 4.33 | 1.80 | 1.41 | 1.67 | 2.96 |  |  |
| Ser-28 | 8.32 | 4.40 | 3.84 |  |  |  |  |  |
| Glu-29 | 8.45 | 4.26 | 2.05  1.91 | 2.25 |  |  |  |  |
| Ala-30 | 8.20 | 4.27 | 1.35 |  |  |  |  |  |
| Val-31 | 7.94 | 3.97 | 1.99 | 0.90  0.81 |  |  |  |  |
| Arg-32 | 8.21 | 4.23 | 1.63 | 1.50 | 3.11 |  |  |  |
| His-33 | 8.29 | 4.73 | 3.19  3.15 |  |  |  |  | 2H 8.25  4H 7.14 |
| Phe-34 | 8.19 | 4.75 | 3.11  2.85 |  |  |  |  | H2/6 7.22  H3/5 7.30  H4 7.27-7.36 |
| Pro-35 |  | 4.37 | 2.24  1.93 | 1.85 | 3.65  3.47 |  |  |  |
| Arg-36 | 8.34 | 4.18 | 1.65 | 1.51 | 3.07 |  |  |  |
| Ile-37 | 8.00 | 4.12 |  |  |  |  |  |  |
| Trp-38 | 8.24 | 4.70 | 3.22  3.11 |  |  |  |  | NH 10.13  2H 7.14  4H 7.61  5H 7.12  6H 7.21  7H 7.45 |
| Leu-39 | 8.00 | 4.23 |  |  |  |  |  |  |
| His-40 | 7.85 | 4.47 | 3.07  2.91 |  |  |  |  | 2H 8.16  4H 7.22 |

**Table S6.**

^1^H, ^13^C and ^15^N chemical shifts for all-*trans* proline conformers of *s*Vpr^25-40^ at 300K in H_2_O:D_2_O (9:1, v/v) at pH 3

|  | HN | Hα | Hβ | Hγ | Hδ | Hε | NH/NH_2_ | Ar-H | Cα | N |
| --- | --- | --- | --- | --- | --- | --- | --- | --- | --- | --- |
| Glu-25 | - | 4.06 | 2.14  2.10 | 2.44 |  |  |  |  | 55.10 | - |
| Leu-26 | 8.71 | 4.39 | 1.60 | 1.60 | 0.89 |  |  |  | 55.29 | 125.50 |
| Lys-27 | 8.52 | 4.31 | 1.82  1.76 | 1.43 | 1.67 | 2.98 | 7.50 |  | 56.49 | 123.69 |
| Ser-28 | 8.28 | 4.39 | 3.83 |  |  |  |  |  | 58.32 | 116.89 |
| Glu-29 | 8.35 | 4.35 | 2.10  1.95 | 2.42 |  |  |  |  | 55.82 | 126.37 |
| Ala-30 | 8.23 | 4.28 | 1.34 |  |  |  |  |  | 52.60 | 125.19 |
| Val-31 | 8.01 | 3.99 | 1.97 | 0.89  0.80 |  |  |  |  | 62.25 | 119.72 |
| Arg-32 | 8.25 | 4.20 | 1.60  1.52 | 1.42 | 3.11 |  | 7.09 |  | 55.99 | 124.87 |
| His-33 | 8.37 | 4.65 | 3.02  3.12 |  |  |  |  | 2H 8.55  4H 7.19 | 54.65 | 120.03 |
| Phe-34 | 8.38 | 4.84 | 2.85  3.14 |  |  |  |  | 2/6 7.24  3/5 7.31  4 7.27 | 55.74 | 123.12 |
| Pro-35 |  | 4.41 | 2.25  1.88 | 1.97 | 3.74  3.56 |  |  |  | 63.04 | - |
| Arg-36 | 8.31 | 4.20 | 1.66  1.52 | 1.45 | 3.06 |  | 7.00 |  | 56.13 | 121.39 |
| Ile-37 | 8.06 | 4.16 | 1.78 | 1.40  1.13  0.82 | 0.82 |  |  |  | 60.86 | 122.01 |
| Trp-38 | 8.34 | 4.73 | 3.25  3.12 |  |  |  |  | NH 10.09  2H 7.18  4H 7.62  5H 7.11  6H 7.19  7H 7.43 | 56.68 | 122.57 |
| Leu-39 | 8.12 | 4.25 | 1.48 | 1.39 | 0.78  0.83 |  |  |  | 55.08 | 124.80 |
| His-40 | 8.19 | 4.54 | 3.15  3.01 |  |  |  |  | 2H 8.50  4H 7.15 | 54.83 | 119.08 |

**Table S7.**

^1^H chemical for amino acids surrounding *cis* proline 35 of *s*Vpr^25-40^ at 300K in H_2_O:D_2_O (9:1, v/v) at pH 3.

|  | HN | Hα | Hβ | Hγ | Hδ | N |
| --- | --- | --- | --- | --- | --- | --- |
| Arg-32 | 8.29 | 4.27 | 1.69  1.51 | 1.60 | 3.12 |  |
| His-33 | 8.41 | 4.60 | 3.12  3.05 |  |  | 120.06 |
| Phe-34 | 8.12 | 4.49 | 2.92 |  |  | 122.37 |
| Pro-35 |  | 3.94 | 1.96  1.90 | 1.75/1.69 | 3.49  3.38 |  |
| Arg-36 | 8.49 | 4.15 | 1.67  1.52 | 1.44 | 3.08 | 121.56 |
| Ile-37 | 8.00 | 4.14 | 1.73 | 1.06 | 0.9-0.6 |  |
| Trp-38 | - | - | - | - | - | - |
| Leu-39 | 8.13 | 4.24 | 1.46 | 1.37 | 0.81  0.77 | 124.80 |

**Table S8.**

Calculated reaction rate constants for the prolyl *cis*/*trans* interconversion of Pro-35 of *s*Vpr^32-38^ based on volume integration of exchange crosspeaks originating from *cis* Hα Pro-35 –*trans* Hα Pro-35 in ROESY experiments with mixing times 200-500 ms

| Mixing time (ms) | 200 | 250 | 300 | 350 | 400 | 450 | 500 |
| --- | --- | --- | --- | --- | --- | --- | --- |
| k_ct_ (s^-1^) | 0.181 | 0.191 | 0.179 | 0.186 | 0.186 | 0.203 | 0.186 |
| Mean reaction rate constant = 0.19 ±0.01 s^-1^ | | | | | | | |


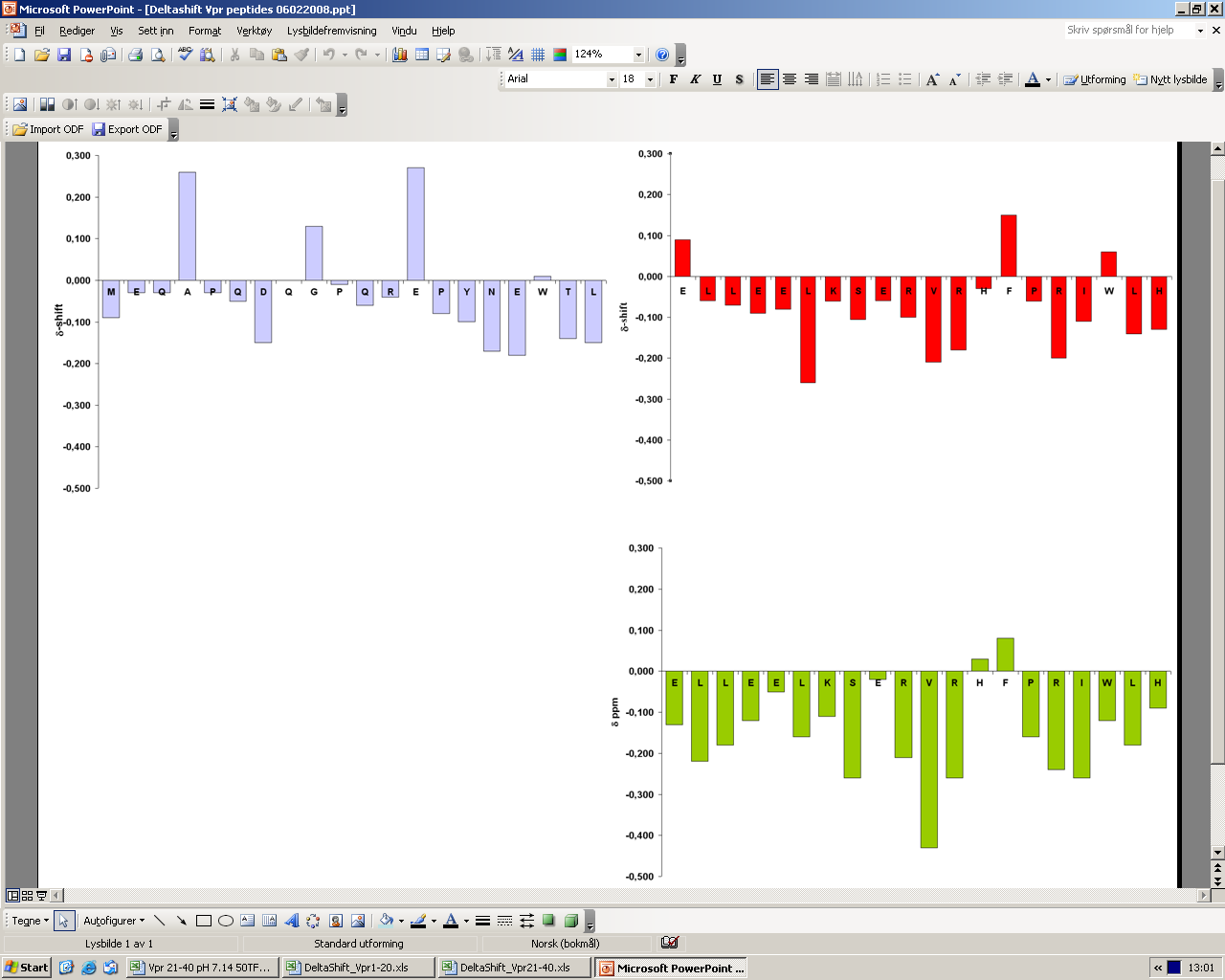


**A**

**B**

**Helix 1**

**C**

**Figure S1.** Chemical shift differences of the Hα-protons between the experimental values and those for random coil residues, for all-*trans* proline *s*Vpr^1-20^ (**A**) and *s*Vpr^21-40^ (**B**) in aqueous phosphate buffer pH 7:D_2_O (9:1, v/v) at 300 K, and *s*Vpr^21-40^ in aqueous phosphate buffer pH 7:TFE-d_2_ (1:1, v/v) at 300 K (**C**).

**A**

**B**

**Figure S2.** Peak intensities of *cis/trans* crosspeaks versus diagonal signal intensities influenced by mixing times observed in the ROESY spectra of *s*Vpr^32-38^. (**A**) *Trans* form diagonal signals. (**B**) *Cis* form diagonal signals.

**
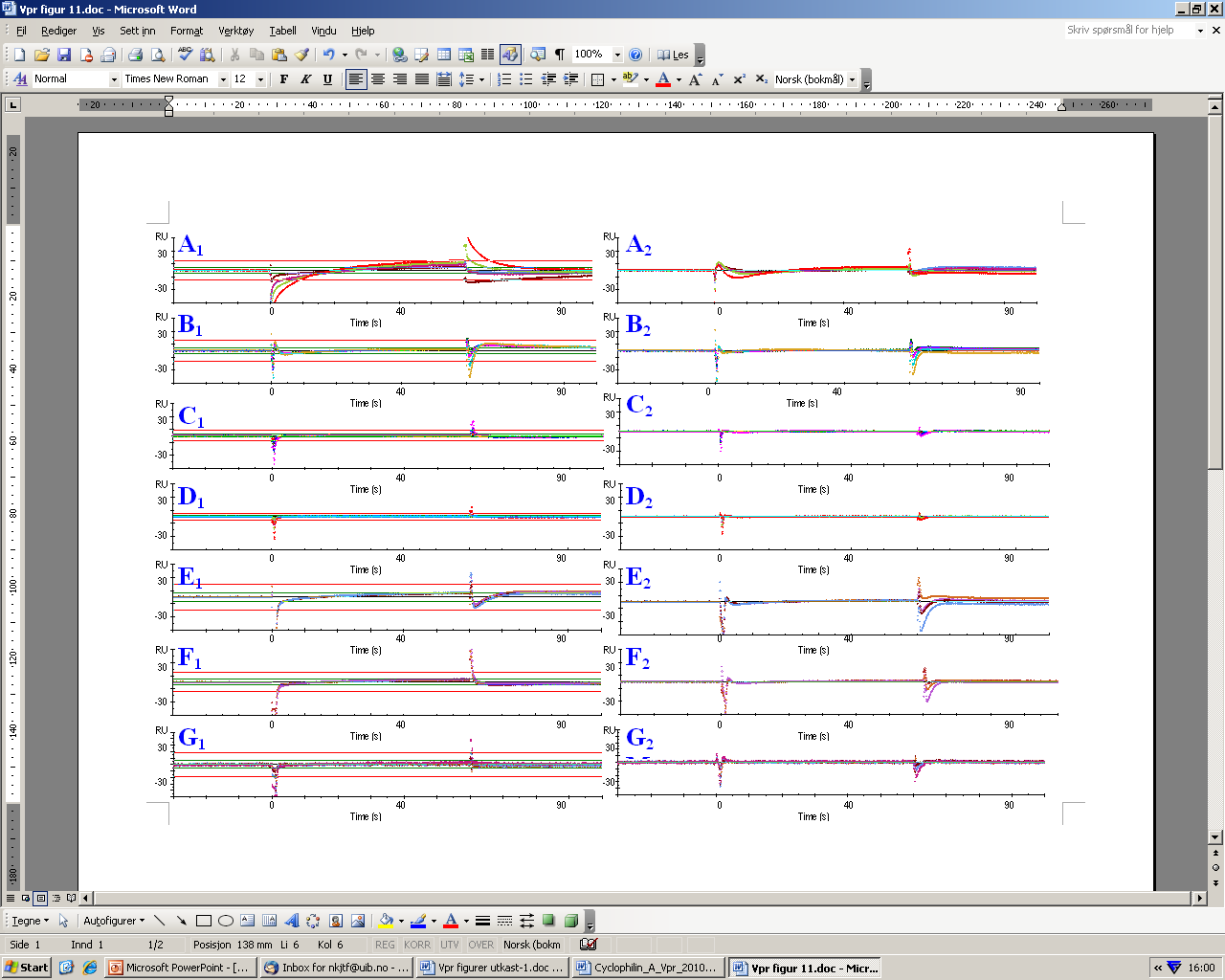
**

**Figure S3.** Optimized fit residuals of SPR sensograms to 1:1 (Langmuir) (**A_1_-G_1_**) and two-state reaction (conformational change) (**A_2_-G_2_**) binding models. The curves originating from the interactions of *s*Vpr^21-40^ (**A**), *s*Vpr^25-40^ (**B**), *s*Vpr^1-40^(**C**), *s*Vpr^1-40^ P5,10,14N (**D**), *s*Vpr^30-40^ (**E**), *s*Vpr^32-38^ (**F**) and *s*Vpr^33-37^(**G**) with CypA were considered to fit to two state reaction model (**A_2_-G_2_**) best.


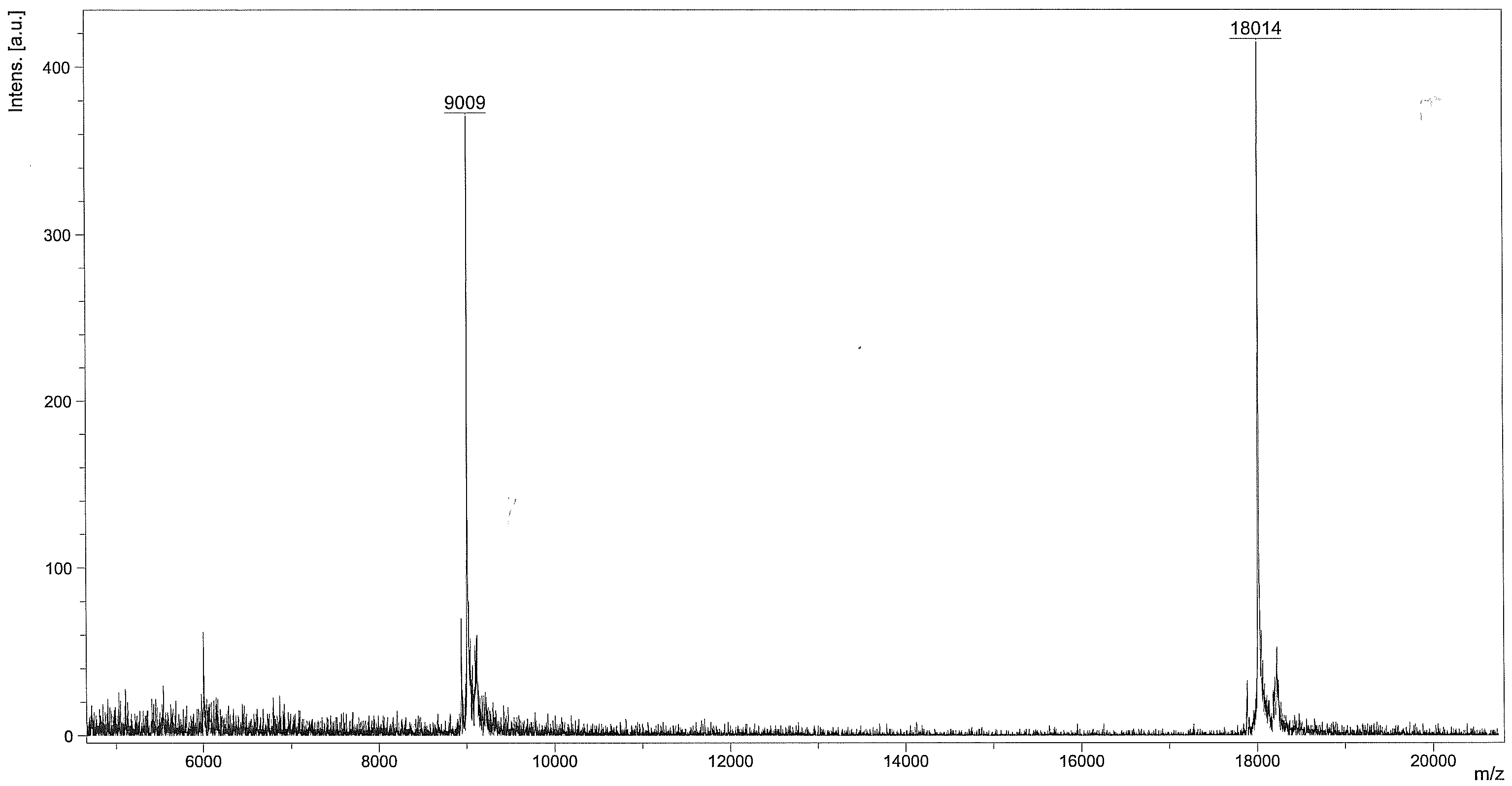


**Figure S4. MALDI-TOF Mass spectrum of CypA.**

Recombinant human CypA was produced and purified to homogeneity.
